# How to explore what is hidden? A review of techniques for vascular tissue expression profile analysis

**DOI:** 10.1186/s13007-023-01109-8

**Published:** 2023-11-19

**Authors:** Karolina Kułak, Natalia Wojciechowska, Anna Samelak-Czajka, Paulina Jackowiak, Agnieszka Bagniewska-Zadworna

**Affiliations:** 1 Department of General Botany, Institute of Experimental Biology, Faculty of Biology, Adam Mickiewicz University, Uniwersytetu Poznanskiego 6, 61-614 Poznan, Poland; 2 Institute of Bioorganic Chemistry, Polish Academy of Sciences, Noskowskiego 12/14, 61-704 Poznan, Poland

**Keywords:** Vascular tissue, Conduit development, Xylem, Phloem, Transcriptomics, Gene expression, Expression profile

## Abstract

The evolution of plants to efficiently transport water and assimilates over long distances is a major evolutionary success that facilitated their growth and colonization of land. Vascular tissues, namely xylem and phloem, are characterized by high specialization, cell heterogeneity, and diverse cell components. During differentiation and maturation, these tissues undergo an irreversible sequence of events, leading to complete protoplast degradation in xylem or partial degradation in phloem, enabling their undisturbed conductive function. Due to the unique nature of vascular tissue, and the poorly understood processes involved in xylem and phloem development, studying the molecular basis of tissue differentiation is challenging. In this review, we focus on methods crucial for gene expression research in conductive tissues, emphasizing the importance of initial anatomical analysis and appropriate material selection. We trace the expansion of molecular techniques in vascular gene expression studies and discuss the application of single-cell RNA sequencing, a high-throughput technique that has revolutionized transcriptomic analysis. We explore how single-cell RNA sequencing will enhance our knowledge of gene expression in conductive tissues.

**Supplementary Information:**

The online version contains supplementary material available at 10.1186/s13007-023-01109-8.

## Introduction

The transition of plants from aquatic ecosystems to land, which occurred over 400 million years ago [1], marked a defining moment in the history of the Earth. This transition was characterized by radical transformations in plant body plans, including the establishment of sporophyte dominance and the development of specific organs such as roots, leaves, flowers, seeds, and vasculature. Successful colonization of terrestrial environments necessitated the evolution of multicellular organs capable of actively penetrating the soil, absorbing and transporting water, nutrients, and signaling compounds crucial for plant growth. The development of efficient conductive (vascular) tissues played a vital role in this process [2]. Vascular tissues are responsible for the transport of water, nutrients, photosynthates, hormones, and vitamins, which are essential for proper plant growth and development [3, 4]. These tissues consist of tube-like cells that facilitate the transport of water and nutrients from roots to leaves (xylem) and the transport of sugars from leaves throughout the plant (phloem) [5]. Vascular tissues exhibit a high degree of specialization and heterogeneity. In addition to their unique functions, they consist of distinct cell types. It is important to note that this heterogeneity is caused that not all cells in xylem or phloem are conductive elements. Phloem includes conductive sieve elements, but also companion cells, phloem parenchyma involved in radial transport and storage and dead fiber cells providing mechanical support. In xylem, conductive (tracheary) elements are only vessels or tracheids, and analogically to phloem, xylem fibers, and parenchyma cells with different functions are present. Tracheary elements in xylem are dead at maturity, while sieve elements in phloem are still-living cells lacking certain organelles, such as nuclei, mitochondria, and plastids [5]. The unique characteristics and heterogeneity of vascular tissue pose significant challenges for molecular analyses, particularly when extracting and analyzing individual cells within the tissue. Bulk molecular analysis using such functionally diverse cellular material leads to inaccurate conclusions. Therefore, analyzing gene expression profiles within vascular tissues requires an understanding of the specific challenges posed by this plant material. The main objective of this work is to select and compare potential methods for analyzing gene expression profiles in conductive cells of plants. Comparative analysis of xylogenesis and phloemogenesis mechanisms is challenging but highly significant. A comprehensive transcriptome analysis of developing vascular tissue is crucial, as the findings could have potential applications in improving wood properties through genetic engineering. Recent years have witnessed a surge in research focused on developing high-throughput techniques, which have greatly accelerated plant molecular science. Scientific investigations are primarily aimed at gaining knowledge about the elusive and unidentified signals that determine vascular tissue differentiation, maturation, and function. However, studying and understanding these complex relationships in plants present a significant challenge that can be addressed through transcriptomic analyses. In this review, our objective is to make strides towards understanding and identifying the best solutions for conducting gene expression analysis in vascular tissues to enhance our understanding of developmental events *in planta*.

## Material selection and analysis

When studying conductive tissues, the selection of appropriate material for testing poses additional challenges. When analyzing the differentiation and development stages of xylogenesis or phloemogenesis, detailed transcriptomic analysis becomes challenging due to high cell variability and the relatively low number of tracheary and sieve elements in whole plant organs. To avoid errors, it is crucial to work with known, anatomically and cytologically controlled material. This can be achieved through histological analyses, along with histochemistry and specific staining based on autofluorescence (e.g., lignin in xylem vessel secondary cell walls), which aids in selecting the proper organ parts.

Observing and studying the anatomical changes during the differentiation of xylem and phloem cells can be achieved through several techniques, including light and fluorescent microscopy. Additionally, serial block-face electron microscopy allows for the study of the surface details of selected plant material (cells or tissue sections). However, none of these methods enable the study of the molecular mechanisms characteristic of the observed cells. In the case of vascular tissue, this becomes particularly problematic due to the deep location of phloem and xylem within plant organs, surrounded by numerous other plant cells.

## Vascular tissue selection and isolation

### Tissue isolation based on anatomical analyses

**Fig. 1:**
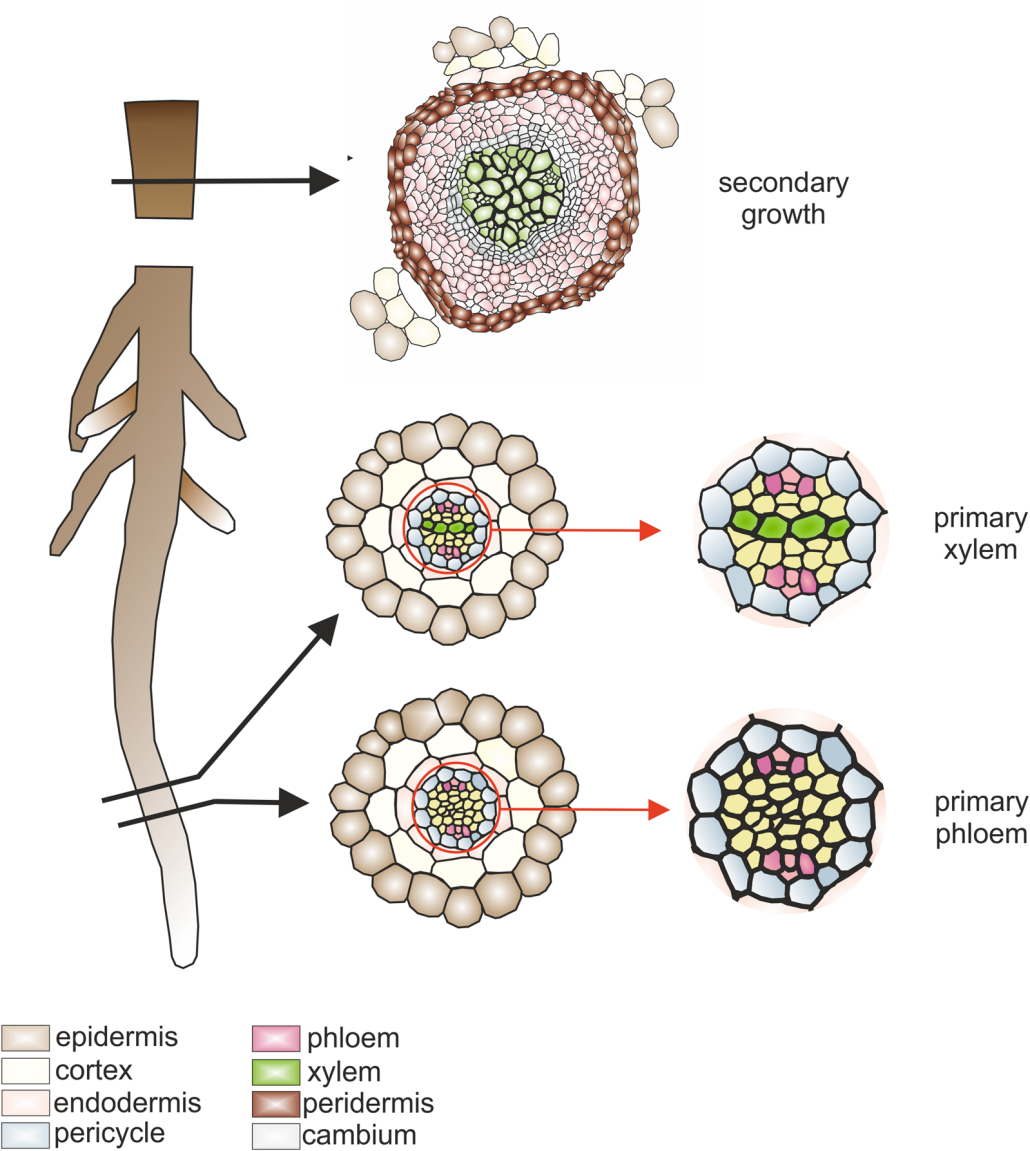
Anatomical screening of root structure and vascular tissue development (drawn not to scale)

Studies on vascular tissue development should always be preceded by precise anatomical analyses of the studied plant organs. Anatomical screening enables the determination of the distance from the root tip, or shoot meristem where phloem and xylem differentiation begins and they mature. It also helps identify the zone with cambial cell formation and the activity of this tissue, which leads to secondary vascular tissue development (Fig. 1). Moreover, this method allows for the identification of differences in the histogenesis of individual organs of a known age, which can strongly contribute to variations in gene expression profiles, as observed during xylogenesis [6]. This approach facilitates the selection of specific organ parts containing tissues at particular stages of development for further analyses aimed at examining gene expression. However, examining gene expression, the result obtained represents a pool of all cells present in the selected organ fragment. If the expression of the tested genes is not tissue- or cell-specific, this approach may not be sufficient to determine the specific tissue in which the expression is detected [7]. To overcome these challenges, the ideal solution is the isolation of specific tissues. In the case of secondary growth, it is possible to manually separate xylem from the surrounding tissues due to its characteristic anatomical structure. However, for other tissues, such as phloem, especially in the primary structure, this approach is not feasible. Nonetheless, other techniques such as Laser Capture Microdissection (LCM) can be incredibly helpful and successfully implemented. The LCM technique is capable of enabling the isolation of individual cells from tissues.

### Laser capture microdissection (LCM)

**Fig. 2:**
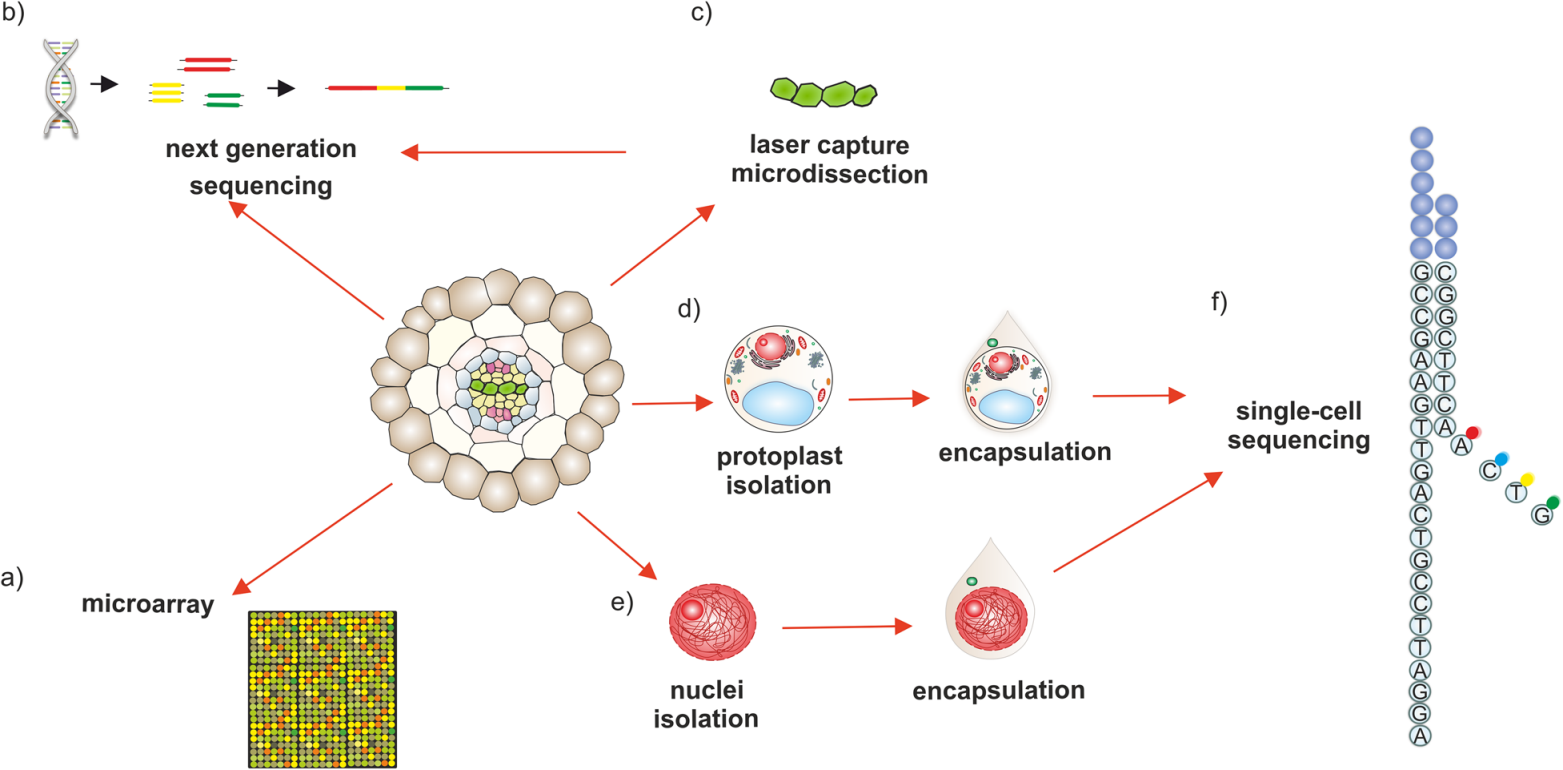
The scheme illustrating methods used for getting and analysing mRNA in the gene expression analysis of vascular tissue cells: **a** microarray; **b** bulk RNA sequencing; **c** laser capture microdissection (LCM); **d** protoplast isolation; **e** nuclei isolation; **f** single-cell RNA sequencing

Laser capture microdissection (LCM) is an alternative method increasingly utilized in plant transcriptomic analysis [8, 9]. LCM has proven to be a valuable technique for transcriptomic analysis of phloem [10] and vascular cambium [11]. It has been employed to select vascular tissue cells in Arabidopsis [12], phloem cells in potato [13], and xylem cells in poplar and eucalyptus [14]. LCM enables the precise isolation of individual cells from complex biological specimens through the use of a laser beam (Fig. 2c). This method ensures the accurate dissection of phloem and/or xylem, and even facilitates the collection of specific cell types from complex tissues, such as phloem or xylem conductive cells, sieve or tracheary elements, respectively. These isolated cells can be further used for cellular and tissue-specific transcriptomic analyses, including microarrays, RNA-seq, and RT-qPCR (Reverse-Transcriptase quantitative Polymerase Chain Reaction).

Combining LCM with anatomical analysis allows for the selection of organ fragments that correspond to specific stages of vascular tissue development (Fig. 1), which may then be collected for RNA isolation and subsequent molecular analyses. LCM is typically followed by RNA isolation and genomic or transcriptomic analyses [15, 16]. Although LCM offers a reliable tool for isolating plant tissues, the preparation of material for this technique and subsequent RNA isolation can be time-consuming and may significantly affect the yield and quality of the isolated RNA. The technique involves several key steps, with sample preparation being one of the most crucial. There are several protocols available for isolating specific cells using LCM, including cryosectioned [17], paraffin-embedded [18, 19], and Steedman’s wax-embedded materials [20, 21]. The utilization of cryosections generally results in higher-quality RNA, while embedding the material preserves the anatomical structure, thereby facilitating the identification of individual cells in complex biological specimens [22–24].

In plants, the LCM technique coupled with high-throughput gene expression profiling is a powerful tool widely used to identify a variety of molecular mechanisms related to vascular tissue [13, 14, 24, 25]. However, it can present challenges in obtaining high-quality RNA for transcriptomic analysis. The material prepared using LCM can be collected and used for further analyses, including RNA isolation and transcriptomic techniques such as microarray or RNA-sequencing.

### Protoplast isolation

Protoplasts are single isolated metabolically active part of the plant cell, without cell wall, what makes them called naked cells. Protoplast isolation technique has had quite a long history dating back to the late 19th century when Hanstein introduced the term *protoplast* for the first time (1880) and Klercker used the very first mechanical method to protoplast isolation (1892). However, the greatest development of protoplast isolation occurred in 1960 when the first enzymatic method for the cell wall removal has been introduced by Cocking. Up to this time, protoplasts have been used extensively in transcriptional studies including vascular tissue. As vascular tissue cells are underrepresented cell types in plant tissues, coupling protoplast isolation with FACS (Fluorescence-Activated Cell Sorting) has become powerful tool for this tissue analyses. Protoplast isolation followed by FACS leads to enrichment of target positive cells and eliminates unwanted negative cells, what is crucial regarding number of phloem and xylem cells in plant tissue. Combining FACS with microarray analysis allowed to describe expression profiles of vascular tissue as well as root developmental zones in Arabidopsis [26]. Protoplasts (Fig. 2d) have been used in phloem and xylem cell transcription analysis by single-cell RNA sequencing (Fig. 2f) of Arabidopsis root [27–30], identification and analysis of xylem in poplar [31], metaxylem in rice root tip [32] or vascular cells in maize shoot stem-cell niche [33]. Using protoplasts has made possible even identification and analysis of particular phloem pole cells in Arabidopsis root [34]. However, with primary xylem cells, only differentiating tracheary elements can be analyzed, since mature elements are dead. Similarly in secondary xylem, only undifferentiated and xylem parenchyma cells are alive.

On the other hand, using protoplasts as biological entities for analyzing gene expression may be puzzling because of particular cell type resistance for cell wall digestion, as well as effect of protoplasting procedure on gene expression and bias en route toward sequencing of smaller-sized cells/protoplasts. All these inherent issues are even more imposing in case of phloem and immature xylem cells which are big-sized with thick secondary cell wall.

### Vascular cell culture systems

The investigation of xylem and phloem cells has become significantly easier with the development of vascular cell culture systems. One such system was established in 1980 by Fukuda and Komamine, who discovered a method for transforming mesophyll cells from *Zinnia elegans* leaves into xylem tracheary elements through auxin and cytokinin induction [35]. This system has proven to be an efficient model for biochemical and physiological studies and has been particularly useful in investigating tracheary elements, which can account for up to 80% of nearly homogenous xylem tracheary elements [36]. The zinnia system has been employed to investigate numerous gene expression profiles, especially genes associated with xylem formation, including programmed cell death (PCD) with protoplast degradation and secondary cell wall formation [37–39]. Additionally, the zinnia system has been used in studies focusing on genes involved in the regulation of tracheary element differentiation [40], patterned secondary cell wall deposition [41], lignin biosynthesis, and genes involved in PCD process such as encoding proteases, lipases and nucleases [38]. Using the zinnia system has also led to the characterization of a group of transcription factors encoded by HD-ZIP III homeobox genes that are crucial for vascular development [42, 43]. Additionally, this system has also revealed the signaling function of xylogen during xylem differentiation [44], and the function of CLE peptides in vascular cell differentiation [45, 46]. One of the first identified genes involved in PCD during tracheary element development was *ZEN1* (*ZINNIA ENDONUCLEASE 1*), which is an S1-type nuclease that functions directly in the degradation of nuclear DNA [47] and is probably localized to the vacuole [48]. In addition to *ZEN1*, several other genes were identified in zinnia cell culture, including the one encoding cinnamyl alcohol dehydrogenase and peroxidase [49, 50], β-tubulin [51], and Tracheary Element Differentiation markers (TED 2–4) [52–54]. Zinnia cell culture has not only served as a source of information about genes related to vascular tissue development but has also been helpful in defining the role of auxin in cell differentiation into tracheary elements [55] and has indicated the presence and possible function of other phytohormones such as brassinosteroids [56], gibberellic acid, and ethylene [57].

In addition to the Zinnia cell culture, Arabidopsis cells can also be induced to differentiate into tracheary elements, albeit with much lower efficiency (up to 30%) [58]. Experiments with Arabidopsis cells transdifferentiated into tracheary elements revealed that genes encoding some microtubule-implicated proteins (*MAP70*) are involved in secondary cell wall synthesis during xylem development [59]. Investigation of Arabidopsis cell culture has also led to the identification of *VND6*, *VND7*, and *SND1* genes as the main regulators of the differentiation of metaxylem and fiber cells [60, 61].

Arabidopsis cell transdifferentiation has created another powerful system for investigating xylem development called VISUAL (Vascular cell Induction culture System Using Arabidopsis Leaves), which reveals the successive differentiation of sieve-element-like cells. Cells in this culture system can also differentiate into xylem and phloem in the presence of bikinin, auxin, cytokinin, and aphidicolin [62]. Using the VISUAL system, several genes involved in xylem and phloem development have been identified, such as *BRI1-EMS-SUPRESSOR1* (*BES1*) [63], and *BZR1*, which is the closest homologous gene to *BES1* with a similar function in promoting the differentiation of vascular stem cells into both xylem and phloem [64].

Moreover, Arabidopsis mutants with abnormal phenotypes in vascular tissue are powerful tools for investigating genes involved in xylem and phloem development. There is a huge collection of Arabidopsis mutant lines that have been helpful in identifying a number of genes involved in vascular tissue development, such as *HD-ZIP III* and *KANADI* genes [42, 65], or the *APL* (*ALTERED PHLOEM DEVELOPMENT*) gene involved in xylem-phloem switching [66]. Other genes identified using Arabidopsis mutants include *VND6* and *VND7* (*VASCULAR-RELATED NAC-DOMAIN6* and *-DOMAIN7*), which regulate the differentiation of xylem vessels [61], *IRX* (*IRREGULAR XYLEM*) genes involved in cellulose synthesis [67], *SMXL3-5* (*SUPPRESSOR OF MAX2 1-LIKE3-5*), which are key regulators of phloem formation [68], *BRX* (*BREVIS RADIX*) with activity in developing protophloem [69], *OPS* (*OCTOPUS*) which regulates phloem differentiation [70], *CLE25/26/45* (*CLAVATA3/EMBRYO SURROUNDING REGION*) genes that encode suppressors of protophloem differentiation [71, 72].

## High-throughput techniques in vascular tissue analysis

High-throughput gene expression profiling has emerged as a major and powerful approach in molecular biology over the past two decades. Unlike traditional gene expression methods such as Northern blot analysis or ribonuclease protection assay, high-throughput techniques enable large-scale gene expression studies with significantly higher sensitivity. Furthermore, the datasets generated from these studies, deposited in repositories, are expected to serve as central resources for the scientific community for many years.

RNA sequencing (RNA-seq) of bulk RNA is the method of choice for analyzing pooled cell populations or specific tissue transcriptomes. This method provides general knowledge and average gene expression patterns for all tested cells or tissues, offering a broad overview of gene expression in the analyzed material. However, performing total RNA-seq may yield average gene expression values for tissue samples containing multiple cell types, potentially overlooking important differences between cells. In the case of xylem and phloem, and their cellular heterogeneity, bulk RNA-seq may be insufficient to obtain information about tissue differentiation and development mechanisms. Moreover, vascular conductive cells (sieve and tracheary elements) are often outnumbered by parenchyma cells, and these average gene expression levels may not accurately represent phloem and xylem conductive cell transcriptomics. This is why one of the methods described earlier should be applied (see “Laser capture microdissection (LCM)” section). Transcriptome analysis of a large group of genes under identical conditions, coupled with knowledge about gene expression profiles, is crucial for understanding the molecular pathways regulating the development of specific organs or tissues in all organisms, including plants and their vascular tissues. The comprehensive characterization of gene expression is a powerful tool for revealing gene expression profiles in specific regions, tissues, and cells within complex multicellular organisms. Given the inadequate nature of our current knowledge in this fundamentally important area, there is a strong motivation for intensive research using high-throughput techniques to study gene expression in exceptional tissues such as xylem and phloem, and their conductive elements.

### Microarray

The first widely used high-throughput technology for simultaneously determining the transcription levels of hundreds to thousands of genes in a short time was microarray analysis (Fig. 2a) [73]. This technique, coupled with fluorescence-activated cell sorting, has been employed to identify and characterize cells and tissues based on their characteristic expression profiles, such as *Arabidopsis thaliana* root cells, including all types of xylem and phloem [26]. The core principle of this technology relies on DNA hybridization, following the Watson–Crick rules, using an array plate with numerous microspots in which short oligonucleotide probes are immobilized [74]. In the initial step, isolated mRNA is reverse-transcribed and then fluorescently labeled using different dyes for control and experimental (treated) samples. Microarray analysis has been a powerful and systematic approach frequently used to investigate gene expression in plants, particularly in the study of vascular tissues, such as xylem [75–77]. However, due to several limitations, microarray technology is being increasingly replaced by other high-throughput gene expression methods. This is primarily due to the limitations of microarrays in analyzing unknown genes, as well as difficulties in detecting multiple transcripts formed by alternative splicing [78].

The microarray technique has enabled the identification of several genes involved in secondary cell wall synthesis in Arabidopsis cell culture. These genes were induced by both SND1 and VND6 gene regulators [79] and belonged to the MYB transcription factor family (MYB46, MYB83, and MYB103), indicating their potential involvement in the process of secondary cell wall formation in xylem vessels and fibers. Furthermore, SND1 and VND6 upregulated several genes involved in cytoskeleton assembly, including genes encoding myosin, tubulin, kinesin proteins, and vesicle transport-related genes (Rab GTPases) [79]. These findings suggest that cytoskeleton rebuilding and vesicle transport are involved in the differentiation of xylem vessels and fibers [79]. In addition, the microarray experiment identified several xylem-specific genes associated with cell protoplast degradation, induced by VND6. These genes include cysteine proteinases (*XCP1*, *XCP2*, *XSP1*), nuclease (*RNS3*), metacaspase (*ATMC9*), and *lipase* (*DSEL*) [79].

Microarray analysis also revealed upregulated genes in *Arabidopsis thaliana* xylogenic cell cultures that are involved in secondary cell wall synthesis and cell death, such as *IRREGULAR XYLEM 3* (*IRX3*), *IRX6*, *IRX8*, *XCP1*, and *XCP2* [80]. This analysis of gene expression in the cell system also identified several NAC domain transcription factors that function as regulators of tracheary element differentiation [61]. Other xylem-specific genes, including *XYLEM CYSTEINE PROTEASE 1* (*XCP1*) and *LOB DOMAIN-CONTAINING protein 15* (*LBD15*), were also identified through microarray analysis [61, 81]. In *Populus trichocarpa*, several *NAC* genes homologous to Arabidopsis ones (*VND*, *NST*, and *SMB*) were found to be functional transcription factors in developing xylem, as revealed by microarray analysis. Many NAC domain protein-encoding genes are suggested to regulate wood formation in poplar, and these genes likely have conserved functions between poplar and Arabidopsis [82]. Additionally, microarray analysis identified genes involved in cell death, cell wall synthesis, cellulose synthase, lignification, structural proteins, cytoskeleton organization, signal transduction, and transcription factors that may be involved in xylem development [82]. Furthermore, microarray analysis demonstrated that auxin plays an essential role in early xylem differentiation by promoting the transition from stage 1 to stage 2 of tracheary element differentiation [55]. Microarray analysis has also been instrumental in exploring phloem development. *BES1* and *BZR1* were found in microarrays as genes involved in the regulation of phloem differentiation [64]. Microarray analysis also revealed an interesting interaction between secondary cell wall synthesis and phloem development [83]. It has provided valuable insights into the molecular basis of phloem cell development in flax stems, which are exceptionally long, and has not only contributed essential information about phloem development, but also holds great potential for future crop improvement [84]. Microarrays have also yielded knowledge about transcription factors involved in phloem formation, such as PEARs (Phloem-enriched Dof transcription factors), which regulate the number of procambium cells dividing into phloem precursors [85].

### Next generation sequencing (NGS)

One of the most advanced molecular methods currently available for gaining detailed information about gene expression in vascular tissue cells is Next Generation Sequencing (NGS), especially when coupled with Laser Capture Microdissection (LCM) (Fig. 2b, c). NGS is often referred to as massively parallel sequencing. Various NGS techniques enable the determination of nucleotide sequences in the entire genome, targeted DNA regions, or RNA fractions in a significantly shorter time compared to the first-generation technology of automated Sanger sequencing [86]. NGS allows for whole-genome sequencing to be completed within a single day, whereas the sequencing of the human genome using the Sanger technique took a decade [87]. The first plant transcriptomes characterized by RNA-seq were rice [88, 89], grapevine [90], and *Arabidopsis thaliana* male meiocyte [91]. Since then, NGS has been widely used in gene expression analysis due to its greater power compared to microarray technology, offering higher sensitivity and wider genome coverage [92]. Moreover, NGS enables the examination of non-coding RNA transcription, which is crucial for cell metabolism and functional regulation. For instance, long non-coding RNAs identified in phloem sap have been investigated using NGS, as they potentially play a role in phloem function [93]. NGS has become a crucial method in the analysis of vascular tissue, allowing for the identification of several genes involved in xylem and phloem development, cell wall biosynthesis, cell division, and protein synthesis/turnover [12]. Notably, NGS has helped identify NAC-domain-containing and MYB transcription factors as key regulators of secondary cell wall synthesis in xylem [94]. Additionally, genes such as *REVOLUTA* (*REV*), *PHLOEM INTERCALATED WITH XYLEM* (*PXE*), *cellulose synthase* *IRREGULAR XYLEM* (*IRX*), and *CLAVATA3/ESR-related* (*CLE*) have been associated with vascular tissue development through NGS analysis [12]. The expression of genes involved in cell division was found to be significantly upregulated in developing vascular tissue [94]. In addition to gene expression analysis, RNA-seq is a valuable tool for studying alternative splicing. For example, a study of xylem transcripts in *Populus trichocarpa* revealed that approximately 36% of the expressed genes in xylem can undergo alternative splicing, including genes related to cell wall synthesis [95].

As RNA-seq has advanced, it has been coupled with LCM to enable gene expression studies at the single-cell level. The RNA-seq-LCM combined technique has been used to characterize transcriptomes of different types of phloem in cucumber (*Cucumis sativus*) fruit during early plant development [96, 97]. This research has been instrumental in phloem studies, leading to the identification of a significant group of phloem-specific transcripts, including 31 genes related to phloem-specific proteins (P-proteins) [96, 97]. Moreover, RNA-seq coupled with LCM has allowed for the investigation of plant responses to various stresses, such as UV radiation, salicylic acid, and other chemical compounds (e.g., antimycin, 3AT, or methyl viologen) that have a substantial impact on vascular tissue [12].

RNA sequencing using NGS technology requires the isolation of total RNA from single cells (in the case of LCM procedure), specific tissue sections, or whole plant organs. Since total RNA-seq provides an average gene expression profile for the cells used in RNA isolation, it is crucial to obtain high-quality RNA to ensure reliable sequencing results. The quality of RNA is assessed using the RNA Integrity Number (RIN), which is crucial for getting high-quality RNA-seq results [98, 99]. However, obtaining high-quality RNA can be challenging, particularly when using LCM, which limits the informativeness of bulk RNA-seq for vascular tissue analysis. This limitation can potentially lead to false results in transcriptomic analysis, particularly when studying phloem and xylem cells, which have distinct RNA characteristics compared to other plant cells.

### Single cell RNA-seq

The development of high-throughput methods for transcriptomic analysis at the single-cell level, known as single-cell RNA-seq (scRNA-seq) (Fig. 2f), has revolutionized our understanding of cell types and states in various biological processes. Within the technological framework proposed by the Plant Cell Atlas Consortium, single-cell sequencing plays a pivotal role in achieving a comprehensive understanding of plant cell structure and function [100]. Among the different approaches, droplet-based solutions have gained popularity. These methods operate based on the following principle: a microfluidic system encapsulates individual cells from a single-cell suspension into nanoliter water-in-oil droplets (Fig. 2d, e), each containing a barcode-tagged bead. This ensures that both the cell and its transcripts receive a unique identifier, enabling unambiguous assignment of transcripts to individual cells. Since the workflow involves oligo(dT) priming, only polyadenylated transcripts are captured and converted into double-stranded DNA libraries. Subsequently, the transcriptomic data are sequenced using NGS platforms, and the information is assigned to individual cells. By clustering cells based on similarities in gene expression profiles, cell types can be annotated using known marker genes. Additionally, cells can be arranged along inferred trajectories that represent distinct cell states, enabling the tracking of developmental and differentiation processes. Detailed experimental and *in silico* workflows for scRNA-seq have been reviewed in [101–104]. Although droplet-based methods offer unprecedented throughput, certain sample types may not be compatible with current platforms. This particularly refers to large cells, which cannot pass through the microfluidic channels and thus will not be encapsulated. Consequently, alternative approaches involving the deposition of single cells in multi-well plates have been explored, sacrificing throughput for increased capture efficiency of diverse cell types [104]. Despite being a groundbreaking technique, the full potential of scRNA-seq can only be realized if specific conditions are met, including sample quality and the availability of reference data. Recent publications have outlined and extensively discussed the paradigms for scRNA-seq studies in plants, covering experimental planning and data analysis considerations [101, 105, 106]. A summary of selected themes is provided in Additional file 1.

Since 2019, when the first high-throughput single-cell transcriptomic data for Arabidopsis roots were published [27–30, 107, 108], droplet-based methods have gained popularity in plant research, enabling the study of crucial processes such as signaling [109, 110], development [111, 112], and cell-type-specific responses to abiotic stresses [113, 114]. Several studies have specifically focused on vascular tissue (Table 1). For example, scRNA-seq was employed to investigate the molecular mechanisms underlying terminal differentiation in xylem cells, particularly the effect of VND7 on the developmental trajectory of individual cells in the Arabidopsis root elongation zone. The study revealed that VND7 alone is sufficient to initiate xylem cell differentiation and identified its target genes involved in this switch. Cortex, trichoblast, and endodermis cells were found to be more prone to transforming into maturing xylem cells. Furthermore, overexpression of *VND7*, *MYB46*, or *MYB83*, which encode transcription factors known to participate in terminal differentiation, was associated with distinct processes of vascular development [108]. The power of single-cell RNA-seq (scRNA-seq) has been harnessed to investigate cytokinin signaling in the Arabidopsis root apical meristem. Precise control of active cytokinin flux from xylem to procambium and phloem cells is crucial, as elevated levels of this phytohormone can disrupt vascular differentiation. By utilizing cell-type-specific transcriptomic data, increased expression of *CYTOKININ OXIDASE3* (*CKX3*) was identified in procambium cells, suggesting a mechanism for cytokinin level regulation in root tissues [110]. Similarly, scRNA-seq coupled with live imaging was employed to examine root phloem development in *Arabidopsis* at single-cell resolution. The study described 19 developmental stages, from stem cell to enucleation, and identified critical genes involved in the entire process. Among these genes were *PHLOEM EARLY DNA-BINDING-WITH-ONE-FINGERs* (*PEARs*) and *APL*, which acted in conjunction with the prevalent PLETHORA regulators to orchestrate phloem maturation [115]. Additionally, scRNA-seq has facilitated the generation of a comprehensive atlas of phloem pole differentiation in Arabidopsis roots, encompassing protophloem sieve element lineage, pericycle cells, metaphloem sieve elements, and companion cells. This approach has shed light on previously underrepresented cell types and significantly advanced our understanding of phloem differentiation processes [116].

**Table 1:** Summary of selected scRNAs-seq studies of plant vascular tissue

Organ	Organism	Sample type	Platform	No. of cells	No. of clusters	Annotated cell types	Ref.
Root	*Arabidopsis thaliana*	Protoplasts	Drop-seq	179–989 per sample	11	Trichoblast, atrichoblast/columella, cortex, endodermis, phloem, protoxylem	Turco et al. [108]
Root	*Arabidopsis thaliana*	Protoplasts	Chromium 10× Genomics	9640–11,313 per sample	34	Procambium, cortex differentiating, cortex initials, endodermis (initials, early, late), epidermis initials, epidermis, trichoblast, atrichoblast, vascular initials, lateral root cap, pericycle, phloem procambium, phloem companion cells, phloem initials, phloem sieve elements, xylem initials, metaxylem, protoxylem, dividing cells, columella, initials	Yang et al. [110]
Root	*Arabidopsis thaliana*	Protoplasts	Smart-seq2	1242	14	P1Δ, P1D, P1 (*PEAR1* reporter lines), CD (*CVP2* reporter line), N57 (*NAC057* and *CALS7* reporter line), CALS7 (*CALS7* reporter line), N73 (*NAC073* reporter line)	Roszak et al. [115]
Root	*Arabidopsis thaliana*	Protoplasts	Chromium 10× Genomics	10,204	15	Phloem sieve element, phloem pole pericycle, metaphloem sieve element, companion cells, pericycle, early phloem, outer layers	Otero et al. [116]
Leaf	*Arabidopsis thaliana*	Protoplasts	Chromium 10× Genomics	5230	19	Mesophyll, epidermis, hydathode, guard cells, bundle sheath, companion cells, xylem parenchyma, phloem parenchyma, procambium cells with features relating to xylem or phloem differentiation respectively	Kim et al. [117]
Cotyledons	*Arabidopsis thaliana*	Protoplasts	Chromium 10× Genomics	14,117	10^a^	Bundle sheath, mesophyll, phloem parenchyma, hydathode, companion cells, guard cells, epidermis, xylem parenchyma, sieve element, procambium cells with features relating to xylem or phloem differentiation respectively	Liu et al. [118]
Stem	*Populus alba* var. pyramidalis	Protoplasts	Chromium 10× Genomics	3170–3626 per sample	20	Xylem (fiber and vessel), xylem mother cells, xylem parenchyma, cambium region, photosynthetic cells, companion cells, phloem parenchyma, phloem mother cells, sieve element, cortex/endodermis initial cells, cortex/endodermis, cork, endoderm, epiderm	Chen et al. [119]
Stem	*Populus alba* × *Populus glandulosa*	Protoplasts	Chromium 10× Genomics	9798	12	Xylem precursor cells, fiber cells, vessels, ray parenchyma	Li et al. [31]
Shoot apex	*Populus tremula × Populus alba*	Nuclei	Chromium 10× Genomics	8324	18	Shoot meristematic cells, mesophyll, trichomes, vascular cells, companion cells, proliferating cells, epidermis, ground meristem	Conde et al. [120]
Stem	*Populus trichocarpa*	Protoplasts	Not specified	12,466	12	Vessel cells, ray cells, phloem, cambium, fiber cells	Xie et al. [159]
Stem	*Populus trichocarpa*, *Eucalyptus grandis*, *Trochodendron aralioides*, *Liliodendron chinense*	Protoplasts	Chromium 10× Genomics MARS-seq2.0	4705 (*P. trichocarpa*)^b^ 5494 (*E. grandis*) 1993 (*T. aralioides*) 2977 (*L. chinense*)	10	Vessel element, fusiform intermediate precursor, ray organizer, fusiform early precursor, ray precursor, fusiform organizer, libriform fiber, ray parenchyma	Tung et al. [121]

^a^For t-SNE, 11 clusters for UMAP

^b^Only cells that were used for clustering are included

Other scRNA-seq discoveries have focused on vascular tissue in Arabidopsis leaves [117] and cotyledons [118]. In the case of leaves, an atlas encompassing all cell types within the vascular tissue, including the less-studied phloem parenchyma cells, was constructed. This atlas provided insights into cell lineage relationships and suggested that phloem parenchyma cells, in addition to their role in transporting sugars and amino acids, are involved in hormone and glucosinolate biosynthesis [117]. The study of cotyledons revealed the early developmental trajectory of vasculature and identified CYCLING DOF FACTOR 5 (CDF5) and REPRESSOR OF GA (RGA) as potential transcription factors involved in regulating this process [118].

Recent years have witnessed several reports on vascular tissue in woody plant stems, primarily in poplar, as well as other angiosperm species. For instance, an atlas of differentiating xylem in hybrid poplar, encompassing vessel cells, fiber cells, ray parenchyma cells, and xylem precursor cells, was constructed. This facilitated the identification of candidate transcription factors, including PagSND2, PagMYB42, and PagSND1, implicated in controlling xylem cell fate [31]. Moreover, scRNA-seq profiling of highly lignified poplar stems not only corroborated existing knowledge of the transcriptomic landscape and development of xylem and phloem but also revealed new insights into phytohormone signaling during vascular differentiation. Additionally, this approach demonstrated the ability to dissect the functional redundancy of paralogs [119]. All the aforementioned studies were performed using protoplasts as the sample type. Further elucidation of woody plant vasculature maturation was made possible through the application of single-nuclei RNA-seq (snRNA-seq) to analyze the poplar shoot apex. By comparing transcriptional landscapes of primary vascular tissue in poplar and Arabidopsis, regulatory genes involved in vascular development were characterized. This analysis unveiled both conserved and unique mechanisms, paving the way for further investigations into vascular tissue differentiation in annual and perennial plants. The scRNA-seq data also facilitated the selection of a gene for detailed functional studies, leading to the delineation of the role of *LAX2* in promoting xylem differentiation in poplar [120]. A recent study combining scRNA-seq and LCM has provided a comprehensive understanding of xylem development in an evolutionary context, spanning from magnoliids through basal eudicots to core eudicots (*Trochodendron aralioides*, *Liliodendron chinense*, *Eucalyptus grandis*, *Populus trichocarpa*). This study presented evidence of a high degree of conservation in ray lineages and greater evolutionary variability in fusiform lineages, with ray parenchyma cells and axial xylem elements following separate developmental paths [121]. Hence, the cited examples leave no doubt that scRNA-seq is a promising technique that can significantly contribute to addressing long-standing questions regarding the key aspects of the structure, development, and function of vascular tissue. Ongoing technological advancements lead us to anticipate that scRNA-seq will soon be effectively complemented by methods simultaneously exploring multiple modalities such as chromatin accessibility or proteomics [122]. Moreover, spatial transcriptomics, which reveals single-cell gene expression profiles in their anatomical context, will further expand the possibilities to study rare and hidden cell types. For example, recently a combination of high-resolution microscopy with spatial transcriptome led to the discovery that phloem and xylem cells are produced from two separate pools of procambium-like cells that display distinct localizations within the secondary vascular tissue of poplar stem [123].

### Transcriptomic validation of selected key genes

Genes identified in all types of transcriptomic analyses need to be further investigated using molecular approaches. RT-qPCR is the method of choice for evaluating the expression profiles of selected genes. Typically, at least three biological replicates are prepared to confirm differential transcript expression, as is customary for a particular cell, tissue, or plant organ at a specific developmental stage. An essential aspect of this analysis is to identify at least two or three reference genes with stable expression in the tested tissue, such as *GAPDH* or *ETIF5A* [124], *18S rRNA*, *actin*, *β-tubulin*, *elongation factor 1α*, or *ubiquitin* [125, 126]. RT-qPCR is a valuable technique for confirming cell differentiation in vascular cell cultures, as it allows the examination of marker gene expression levels, for example, *APL* or *SEOR1* for phloem cells and *GSK3S* for xylem cells [62]. Based on this, it is possible to determine whether selected genes are involved in xylem or phloem development as downstream targets of xylem differentiation regulators in cell cultures through their overexpression [79].

Reverse genetics approaches [61] are another validation method for selected genes, particularly for species with a T-DNA mutant library, such as *A. thaliana* [127]. *A. thaliana* possesses an extensive collection of T-DNA mutant lines deposited in the NASC (The Nottingham Arabidopsis Stock Centre), which were initially obtained in 2003 using mutagenesis methods [128]. Several Arabidopsis T-DNA mutants have been utilized in vascular tissue research, including *cs7* (mutation in the *CALLOSE SYNTHASE 7* gene) [129], *clerk-5* (mutation in the *CLE-RESISTANT RECEPTOR KINASE* gene) [130], *aap2* (mutation in the *AMINO ACID PERMEASE 2* gene) [131], *apl* (mutation in the *ALTERED PHLOEM DEVELOPMENT* gene) [66], *ops-2* (mutation in the *OCTOPUS* gene) [70], several mutants involved in xylem secondary cell wall synthesis [132], *vnd7* (mutation in the *VASCULAR-RELATED NAC DOMAIN7* gene) [52], and *cep1* (mutation in *PAPAIN-LIKE CYSTEINE PROTEASE*) [133]. Using Arabidopsis T-DNA mutants has led to the identification of *APL* as a key gene in phloem development [66], the *TDIF–TDR–WOX4* signaling pathway as crucial for maintaining vascular structure during secondary growth [134], and the *TMO5/LHW* transcription factor as an important regulator of vascular development in plant embryos [135].

## Gene/transcript localization and visualization in vascular tissue

### In situ hybridization (ISH)

**Fig. 3:**
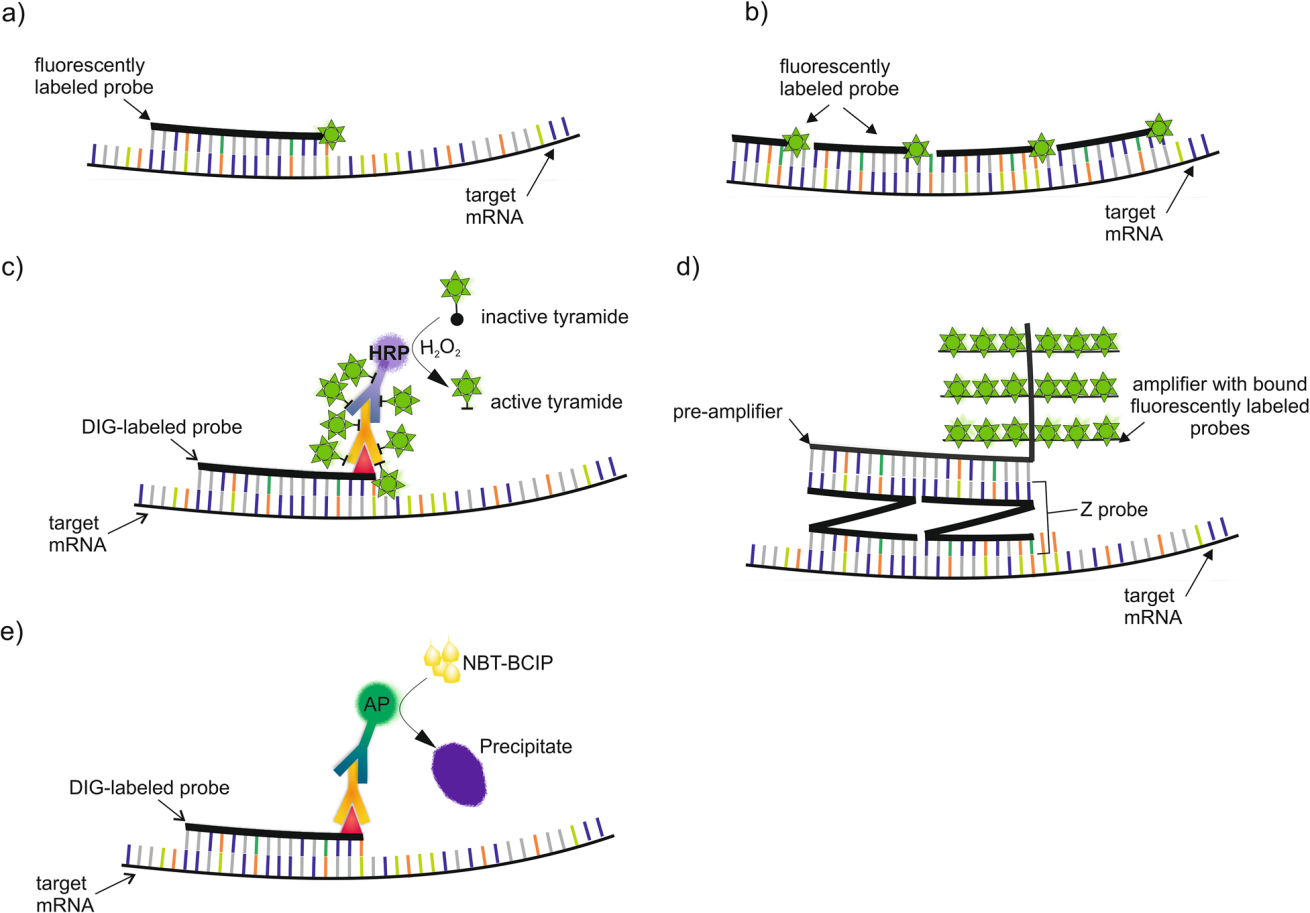
Methods used to visualize mRNA localization based on the hybridization phenomenon **a** direct fluorescent in situ hybridization (FISH); **b** *Stellaris™* FISH; **c** tyramide signal amplification-FISH; **d** RNAscope*-*ISH; **e** alkaline phosphatase-ISH

Significant advances in microscopic techniques have provided valuable tools that enable comprehensive insights into complex vascular tissue development processes by localizing and visualizing even a single copy of a transcript. In situ hybridization (ISH) is a powerful method for investigating mRNA localization, and when combined with bioinformatics tools, it enhances the understanding of gene expression mechanisms [136, 137]. This technique utilizes labeled molecular probes that directly bind to complementary target mRNAs, enabling the observation of transcript localization at the cellular and subcellular level [138]. Initially, ISH employed radioisotope-labeled probes for their high sensitivity. However, due to the high risk of radioactive exposure and the challenges associated with radioactive waste disposal, fluorescently labeled and/or hapten-labeled probes have replaced them [139]. The fluorescent in situ hybridization (FISH) technique is much safer but can be challenging, especially in plant tissues with high autofluorescence, particularly in xylem. This challenge is more pronounced for short transcripts with fewer possible probe binding sites and for those with very low abundance in the cell [140]. Additionally, the presence of native molecules such as lignin, tannins, or chlorophyll contributes to high levels of background autofluorescence. Moreover, autofluorescence can be induced or enhanced by the fixation of plant samples in aldehyde-based fixatives [141]. To enable highly sensitive spatial transcriptomics analysis, several approaches have been introduced (Fig. 3).

### ISH/FISH technique modifications

*The Stellaris™ FISH technique* is a modification of the standard FISH method that enhances the fluorescence signal and minimizes false-negative or false-positive signals resulting from non-specific probe binding or blockage of the probe binding site by proteins interacting with RNA [142]. The Stellaris FISH technology employs multiple singly labeled probes that hybridize along targeted RNA transcripts, collectively emitting significantly higher fluorescence signals than a single probe (Fig. 3b). In addition to mRNA detection, this technology allows for the quantification of individual RNA molecules since each localized transcript is observed as a diffraction-limited spot through conventional fluorescence microscopy [142].

*Tyramide Signal Amplification* (*TSA*), also known as the CAtalyzed Reporter Deposition (CARD) method, is an ultra-sensitive technique that utilizes enzymatic enhancement to increase signal intensity and detect low-abundance mRNA targets [143, 144]. TSA utilizes the catalytic activity of horseradish peroxidase (HRP), which, in the presence of hydrogen peroxide (H_2_O_2_), converts fluorescently labeled tyramide to produce highly reactive tyramide radicals that rapidly bind to tyrosine residues on and proximal to the enzyme site (Fig. 3c) [145]. FISH combined with TSA employs probes labeled with a hapten (usually digoxigenin, but also biotin, fluorescein, dinitrophenol (DNP) recognized by antibodies. In the direct approach, anti-hapten antibodies are tagged with HRP, although more commonly, an indirect approach is used, involving the use of two types of antibodies (anti-hapten and HRP-tagged). TSA detection may provide up to a 100-fold increase in signal compared to conventional fluorescent probes, which is particularly useful in plant tissues where strong autofluorescence makes signal visualization challenging [146].

Another ultra-sensitive method that enables the detection and localization of target mRNA with signal amplification and suppression of background noise is *RNAscope in situ hybridization* [137, 147, 148]. The major difference compared to standard RNA ISH lies in the type of molecular probe used. RNAscope utilizes a pair of Z-shaped probes, each consisting of three fragments: the lower region, which is complementary to the target sequence; the upper region, which forms the binding site for the pre-amplifier; and the intermediate sequence that links them. The pre-amplifier binds to the binding site created by each ZZ pair, allowing for the subsequent attachment of multiple amplifiers. Finally, labeled probes adhere to the binding site on all amplifiers, enabling the visualization of the target mRNA [137]. RNAscope is compatible with both fluorescently labeled probes (Fig. 3d) and various hapten-labeled probes, thereby facilitating the subsequent use of chromogenic and fluorescent dyes [147].

*ISH-AP* is a widely used modification of the standard in situ hybridization technique that overcomes the autofluorescence issue by utilizing alkaline phosphatase (AP) catalytic activity [149, 150]. In this reaction, similar to the TSA technique, a hapten-labeled probe is used and detected with high affinity by anti-hapten antibodies conjugated to alkaline phosphatase (Fig. 3e). Alternatively, unconjugated anti-hapten antibodies and conjugated secondary antibodies may also be used. AP activity is localized by the formation of a purple precipitate resulting from the hydrolysis of 5-bromo-4-chloro-3-indolyl phosphate (BCIP) to 5-bromo-4-chloro-3-indole in the presence of nitroblue tetrazolium chloride (NBT) [151]. The AP activity can also be utilized in RNAscope assays, where AP-labeled probes are used instead of fluorescently labeled probes [147, 152].

## Conclusion

Transcriptomic analysis plays a crucial role in discovering new gene functions and transcription factors, unraveling regulatory mechanisms of gene expression, and identifying factors that influence cell and tissue functionality. While significant knowledge has been gained about xylem development through transcriptomic analysis [153], our understanding of developing phloem cells remains limited. It is known that the MYB transcription factor APL is a key regulator of sieve element differentiation [66]. Some regulatory factors involved in phloem and xylem development have been identified [3], but the mechanism that halts the cell degradation during phloem development is still unknown. Xylogenesis, unlike phloemogenesis, has been extensively studied, such as in the roots of *P. trichocarpa*, where all stages of xylem development and differentiation between different types of roots (pioneer and fibrous roots) have been characterized [154].

While the aforementioned transcriptomic analysis methods have provided valuable insights into vascular tissue and its development, there are still aspects of xylem and phloem development that are challenging to investigate using traditional tissue-based approaches. Obtaining transcriptome information from single cells could be instrumental in addressing these challenges, although it presents its own set of difficulties, such as with LCM, or through the latest high-throughput method known as single-cell RNA sequencing. Single-cell RNA-seq represents a significant advancement in high-throughput analysis of gene expression, emerging only a decade after the advent of total RNA sequencing. When the first single-cell RNA sequencing protocol was published in 2009 [155], it revolutionized research involving low-abundance cells, such as xylem and phloem cells, within whole plant organs. scRNA-seq offers numerous advantages compared to bulk RNA-seq, including the ability to identify rare cell populations, detect mutations in individual cells, and determine cellular heterogeneity within a pool [156]. Initially, scRNA-seq was primarily utilized in human and animal research, and it took several years to apply this technique to plants [30, 157]. However, within a short span, this technique has enabled the discovery of previously unknown elements of plant biology, such as genes regulating lateral root development [158] and regulators of root hair density in response to phosphate levels [111]. Consequently, scRNA-seq holds great promise in fully comprehending the regulation of plant genes.

Nevertheless, total RNA sequencing and other transcriptome analysis techniques, such as microarrays and in situ hybridization, still have their place and remain valuable and relatively straightforward methods for studying plant transcriptomes.

### Supplementary Information

**Additional file 1.** Overview of selected considerations for the application of scRNA-seq for plant studies.

## Data Availability

Not applicable.
