# Supplementary material for: How to explore what is hidden? A review of techniques for vascular tissue expression profile analysis
Source: Plant Methods. 2023 Nov 19;19:129. doi: 10.1186/s13007-023-01109-8 (PMC10659056; doi:10.1186/s13007-023-01109-8)
Supplement: Supplementary file 1 — Additional file 1. Overview of selected considerations for the application of scRNA-seq for plant studies. [file 13007_2023_1109_MOESM1_ESM.docx]

Overview of selected considerations for the application of scRNA-Seq for plant studies

| **sample type** | protoplasts | • representation of the transcriptome fractions accumulated over time in the cytoplasm and recently synthesized in the nucleus; • limited to models amenable to protoplasting; • bias towards easier-to-dissociate cell types; • fragility - protoplast may burst and release RNA that increases noise in sequencing data; • induction of stress in response to handling procedures may affect gene expression and cell clustering accuracy; • fresh samples must be processed |
| --- | --- | --- |
|  | nuclei | • less impact on the transcriptome during sample preparation; • samples can be frozen; • bias towards recently synthesized fraction of the transcriptome; • reduced gene capture per cell; • more debris |
| **number of cells (nuclei)** | 10^4^-10^5^ range | • in-depth insight into cell types; • detection of rare cell types; • available in microfluidics systems with inherent cell size limits - not compatible with all sample types; • high cost |
|  | 10^3^ range | • available in systems compatible with all cell sizes; • good for screening studies; • cost-effective; • detection of most abundant cell types |
| **sequencing depth** |  | • depends on the amount of RNA per cell, which varies greatly across sample types; • 10,000 - 50,000 reads/cell can be sufficient for cell-type classification; • increased depth reduces transcriptional noise, provides detailed information on gene expression |
|  |  |  |
| **identification of marker genes for cell type annotation** | experimental approaches | • classic methods including *in situ* hybridization and reporter systems are low-throughput and laborious; • spatial transcriptomics provides high-throughput single-cell profiling in anatomical context but is only starting to be developed for plants |
|  | computational tools | • cell state transitions should be considered to include developmental-stage-specific markers and capture cluster dynamics; • include reference-based classifiers and data-driven algorithms that rely on differentially expressed genes |
